# Posterior fossa ependymoma in neurodevelopmental syndrome caused by a de novo germline pathogenic *POLR2A* variant

**DOI:** 10.1002/ajmg.a.62869

**Published:** 2022-06-11

**Authors:** Roberto Paparella, Anna Maria Caroleo, Emanuele Agolini, Giovanni Chillemi, Evelina Miele, Lucia Pedace, Martina Rinelli, Simone Pizzi, Luigi Boccuto, Giovanna Stefania Colafati, Mariachiara Lodi, Antonella Cacchione, Andrea Carai, Maria Cristina Digilio, Paolo Tomà, Marco Tartaglia, Angela Mastronuzzi

**Affiliations:** ^1^ Department of Maternal and Child Health and Urology Sapienza University of Rome Rome Italy; ^2^ Department of Onco‐Hematology, Cell Therapy, Gene Therapy and Hemopoietic Transplant Bambino Gesù Children's Hospital, IRCCS Rome Italy; ^3^ Translational Cytogenomics Research Unit, Bambino Gesù Children's Hospital IRCCS Rome Italy; ^4^ Department for Innovation in Biological, Agri‐food and Forestry Systems Tuscia University Viterbo Italy; ^5^ Institute of Biomembranes, Bioenergetics and Molecular Biotechnologies National Research Center Bari Italy; ^6^ Genetics and Rare Diseases Research Division, Bambino Gesù Children's Hospital IRCCS Rome Italy; ^7^ School of Nursing, College of Behavioral, Social and Health Sciences Clemson University Clemson South Carolina USA; ^8^ Neuroradiology Unit, Department of Imaging, Bambino Gesù Children's Hospital IRCCS Rome Italy; ^9^ Neurosurgery Unit, Department of Neurosciences, Bambino Gesù Children's Hospital IRCCS Rome Italy; ^10^ Department of Imaging, Bambino Gesù Children's Hospital IRCCS Rome Italy

**Keywords:** ependymoma, germline variant, hypotonia, neurodevelopmental syndrome, *POLR2A*

## Abstract

Ependymoma is the third most common pediatric brain tumor. Predisposition to develop ependymomas has been reported in different hereditary diseases, but the pathogenic variants related to the familial syndromes have rarely been detected in sporadic ependymomas. De novo variants in *POLR2A*, the gene encoding the largest subunit of RNA polymerase II, cause a neurodevelopmental disorder with a wide range of clinical manifestations, characterized by severe infantile‐onset hypotonia, developmental delay, feeding difficulties, palatal anomalies, and facial dysmorphisms. As somatic events, *POLR2A* mutations represent a recurrent somatic lesion in benign meningiomas. Here we describe a case of ependymoma in a 2‐year‐old male with a de novo pathogenic variant in *POLR2A* predicted to impair proper interaction of the subunit with transcription‐elongation factor TFIIS, whose function is required for back‐tracking of the enzyme due to elongation blocks or nucleotide misincorporation, and expected to result in an increased error and reduced elongation rates. To date, ependymoma has never been reported in patients harboring pathogenic *POLR2A* variants. Further information is required to explore the possibility of a differential clinical and functional impact of the pathogenic *POLR2A* variants and the eventual inclusion of the *POLR2A* neurodevelopmental disorder among the cancer predisposition syndromes with the possible development of ependymomas.

## 1 INTRODUCTION

The RNA polymerase II subunit A (*POLR2A*) gene encodes the largest catalytic subunit of the RNA polymerase II enzyme (Wintzerith et al., 1992), which mediates the transcription of all protein‐coding and several non‐coding RNA genes in eukaryotic cells. Mutations in *POLR2A* have originally been reported as somatic events implicated in oncogenesis in 2016, when they were specifically associated with a clinically distinct subset of meningiomas (Clark et al., 2016). More recently, de novo germline variants in the same gene have been reported to underlie a neurodevelopmental syndrome characterized by severe infantile‐onset hypotonia, developmental delay, microcephaly, visual anomalies, feeding difficulties, palatal defects, cardiac and urogenital malformations, and facial dysmorphisms (Haijes et al., 2019). In fact, a complex scenario linking constitutional *POLR2A* mutations to a wide spectrum of phenotypes, possibly related to the differential functional impact of the wide spectrum of mutations, is emerging (Hansen et al., 2021).

Ependymomas are the third most common pediatric neoplasms of the central nervous system (CNS), accounting for approximately 10% of brain tumors in children, with a peak incidence in early childhood, as more than 50% of all cases occur in children under 5 years of age (Kilday et al., 2009; Shu et al., 2007). Pediatric ependymomas are generally intracranial in origin and most commonly arise in the posterior fossa. Thus far, nine subgroups have been described based on DNA methylation profiling (Malbari & Lindsay, 2020). Although their increased incidence has been reported in certain cancer‐prone diseases, such as neurofibromatosis type 2 (NF2), multiple endocrine neoplasia type 1 (MEN1) syndrome, and Turcot syndrome (Campian & Gutmann, 2017; de Bont et al., 2008), they mostly occur as sporadic events with a poorly characterized genetic risk.

Here we describe a child with facial anomalies, cleft lip and palate, developmental delay, hydronephrosis, club feet, who presented with gait ataxia and hydrocephalus due to a posterior fossa ependymoma at 2 years of age. After surgical removal of the tumor, he had a progressively ingravescent neurological condition, and genetic studies documented a previously unreported de novo germline *POLR2A* variant.

## 2 CASE REPORT

**FIGURE 1:**
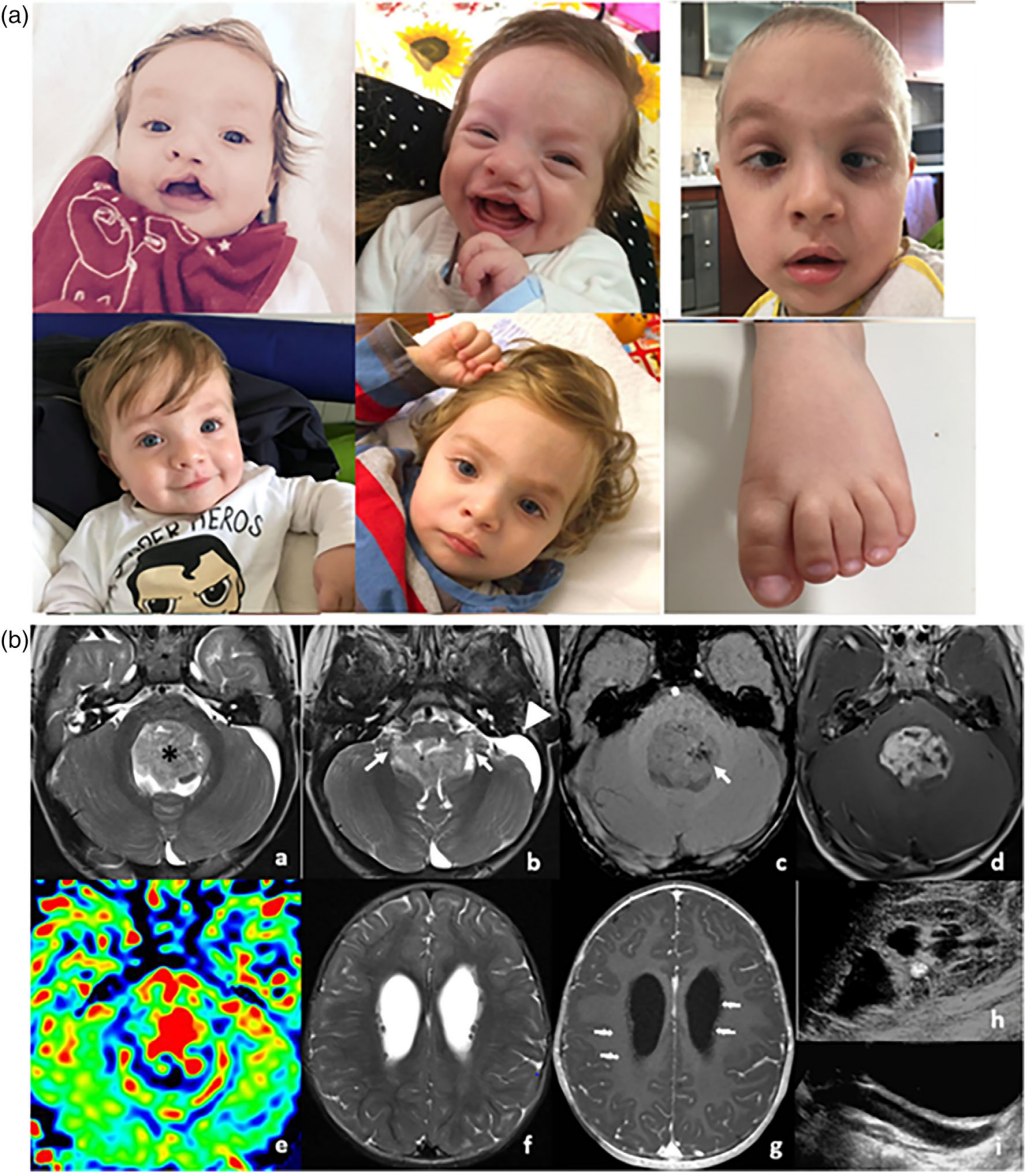
(a) Patient's facial appearance at different ages; left foot with partial cutaneous syndactyly between the 2nd and 3rd toes. (b) Posterior fossa ependymoma. Magnetic resonance imaging (MRI), T2‐weighted (T2w, a, b), susceptibility‐weighted imaging (SWI, c) gadolinium‐enhanced T1‐weighted (GdT1w, d) axial images. Fourth ventricular mass (*) shows heterogeneous T2 hyperintensity (a, b), extension through the foramina of Luschka (b, arrows), punctate hypointense foci (c, arrow) consistent with intratumoral calcifications, predominantly solid enhancement on GdT1w images (d), and elevated perfusion in the cerebral blood volume (CBV) map (e). Note enlargement of the pericerebellar cisternal space on the left with scalloping of the bone, suggestive of an arachnoid cyst (b, arrowhead). Multiple, bilateral periventricular nodular heterotopia. T2w (f) and GdT1w (g) axial MRI images show bilateral heterotopic nodules isointense to gray matter on both T2w and T1w images without contrast‐enhancement, causing distortion of the ventricular margins (arrows). Renal involvement. Ultrasound examination of the right kidney reveals hydroureteronephrosis and lithiasis (h, i)

The proband, a 2‐year‐old white male of Italian ancestry, presented with gait ataxia at the emergency department. He was born at 41 weeks of gestational age, by cesarean section, and his birth weight was 3.110 kg. During pregnancy, cleft lip and hydronephrosis were diagnosed by fetal ultrasound examination. Apgar scores were 9 and 10 at 1 and 5 minutes, respectively. The family history was unremarkable. Clinical examination at 2 years of age showed macro‐dolichocephaly, facial anomalies (sparse and large eyebrows, prominent eyes, elongated palpebral fissures, thick lips, large ears), repaired unilateral cleft lip and palate (surgically corrected at 5 months of age), bifid uvula, bilateral clubfoot, partial cutaneous syndactyly between the second and third toes. Renal ultrasound examination revealed unilateral congenital hydronephrosis (Figure 1a; Table 1). The patient suffered from recurrent upper airway infections. Echocardiography showed late spontaneous closure of patent foramen ovale and patent ductus arteriosus. The baby sat without support at 9 months of age, crawled at 11 months, walked without help at 18 months, and spoke his first words at 22 months. Brain and spine magnetic resonance imaging (MRI) showed obstructive hydrocephalus due to a posterior fossa tumor; therefore, a third ventriculostomy was urgently performed. MRI also detected multiple isointense periventricular nodules, in the absence of restricted diffusion and post‐contrast enhancement, which were described as heterotopia (Figure 1b a–g). He subsequently underwent near‐total resection of the localized tumor, and post‐resection histology proved the tumor to be a classic ependymoma (WHO grade II). He developed postoperative neurological deficits including dysphagia to solids and liquids, sialorrhea, and convergent strabismus. Moreover, he showed a regression in his developmental milestones, gradually losing language, developing trunk and four‐limb hypotonia, and requiring support while walking. He was initially treated with surgery and radiotherapy and subsequently presented with various recurrences of the disease, which were treated with surgery, reirradiation, and chemotherapy.

**TABLE 1:** De novo heterozygous missense variant c.3865G>a (p.Glu1289Lys) of *POLR2A*: Similar and novel clinical features compared to previously reported phenotypes

Organ and tissue systems	Alterations	Previously reported by Haijes et al.^a^	Previously reported by Hansen et al.^b^	Study case [c.3865G>A (p.Glu1289Lys)]
Head and neck	Facial anomalies (≥ 2 dysmorphic features)	10/16	9/12 (facial dysmorphisms and strabismus are considered together)	Sparse and large eyebrows, prominent eyes, elongated palpebral fissures, thick lips, and large ears
	Strabismus	11/15		Convergent strabismus^c^
	Microcephaly––macrocephaly	5/15	3/12	Macro(−dolicho)cephaly
	Orofacial clefts	1/16	0/12	Unilateral cleft lip and palate, and bifid uvula
Cardiovascular	Congenital and acquired heart diseases	1/15	3/12	Late spontaneous closure of patent foramen ovale and patent ductus arteriosus
Respiratory	Respiratory tract diseases	8/15	5/12	Recurrent upper respiratory infections
Gastrointestinal	Feeding difficulties	10/15	7/12	Dysphagia^c^
Urogenital	Functional and anatomical disorders	3/16	7/12	Unilateral hydronephrosis and lithiasis (right kidney) (Figure 1b,h,i), unilateral duplex collecting system (left kidney)
Musculoskeletal	Abnormalities	7/15	5/12	Bilateral clubfoot and kyphosis
CNS/neurodevelopment	Brain MRI abnormalities	12/16	7/12	Heterotopia and obstructive hydrocephalus
	CNS tumors	0/16	0/12	Ependymoma
	Developmental delay	15/15	12/12	Motor and speech‐language delay
	Developmental regression	4/15	4/12	Loss of language; very slow progress in all areas of development
	Intellectual disability	5/15	8/12	Moderate intellectual disability
	Hypotonia	14/15	8/12	Trunk and four‐limb hypotonia ^c^
	Ataxia	0/15	7/12	Gait ataxia
	Behavior/autism spectrum disorder	6/15	6/12	Behavior (and attention) disorder
	Disturbed sleeping	7/15	5/12	Pavor nocturnus, central sleep apnea

^a^ One pregnancy was terminated because of corpus callosum agenesis, frontonasal dysplasia, and a cleft lip; postmortem examination showed additional alterations.

^b^ One individual, previously published by Haijes et al. has been described due to available supplementary information.

^c^ Postoperative neurological deficits.

A neurodevelopmental evaluation, performed at the age of 5 years using the Griffith Mental Development Scales, 3rd Edition and the Vineland Adaptive Behavior Scales, 2nd Edition––Survey Interview Form, revealed significantly lower than normal for age psychomotor development and adaptive function. At the last follow‐up evaluation, at the age of 6 years, he showed cognitive deficits with developmental, language and speech delay (impaired comprehension and expressive language, inability to produce intelligible speech sounds), and attention and behavioral disorder. Physical examination revealed, in addition to the abovementioned dysmorphic features, diffuse hypotonia and dorso‐lumbar kyphosis. He reached good head control, despite an abnormal head posture due to strabismus, and decent trunk control in sitting, albeit with some sudden losses of balance. He had an unsteady gait, needing bilateral ankle‐foot orthoses when standing and walking. Unfortunately, after a few months the patient died from a new recurrence.

## 3 GENETIC TESTING

Clinical investigations and genetic analyses were conducted in accordance with the Helsinki Declaration, after obtaining informed consent from the patient's parents for the genetic testing. Array comparative genomic hybridization tests of the proband and parents did not detect any clinically or functionally relevant rearrangement. Parallel sequencing analysis directed to identify the occurrence of somatic cancer‐associated gene fusions (rearrangement involving the following genes: *ALK, BRAF, BRD3, BRD4, CAMTA1, CCNB3, CIC, EGFR, EPC1, ERG, ETV6, EWSR1, FGFR1, FGFR2, FGFR3, FOXO1, FUS, GLI1, HMGA2, MAML2, MET, MYB, MYBL1, MN1, YAP1, NCOA2, NOTCH1, NOTCH2, NTRK1, NTRK2, NTRK3, NUTM1, PDGFB, PDGFRA, PDGFRB, PIK3CA, PLAG1, PRKCA, RAF1, RELA, RET, ROS1, SS18, STAT6, TAF15, TERT, TFE3, TFEB, TFG, USP6, YWHAE, VGGL2*) in the neoplastic lesion was negative. Similar negative results were obtained by performing a mutation scan on formalin‐fixed paraffin‐embedded (FFPE) tumor and matched peripheral blood specimens using custom‐designed NimbleGen SeqCap probe hybridization (Roche NimbleGen), considering a custom panel covering major pathways implicated in oncogenesis. The FFPE material was also analyzed for DNA methylation profiling using the Infinium EPIC array (Illumina), as previously described (Cacchione et al., 2021). Generated methylation data were compared to the Heidelberg brain tumor classifier 11b43 to assign a subgroup score for the tumor compared to 91 different brain tumor entities (Capper et al., 2018). The DNA methylation profiling confirmed the histological diagnosis. The primary tumor clustered in the class “ependymoma, posterior fossa group A (EPN_PF_A)”, with optimally calibrated scores (>0.9) (Supplementary Table S1). This approach was also used to explore the occurrence of copy number variation, which did not document functionally/clinically relevant structural rearrangements (Supplementary Figure S1). Trio‐based clinical exome sequencing, performed on genomic DNA obtained from leukocytes (Twist Bioscience, South San Francisco, CA), did not reveal any cancer‐associated pathogenic variant, but revealed a novel de novo *POLR2A* missense variant, c.3865G > A (p.Glu1289Lys). Confirmation by Sanger sequencing was not performed due to the high quality of the POLR2A variant call. This variant had not previously been reported in public databases (GnomAD), was predicted to be probably damaging by multiple in silico tools, had a CADD score of 29.8, and was classified as a likely pathogenic according to the ACMG criteria (PP3, PM1, PM2, and PS2). The variant was submitted to the LOVD database (https://databases.lovd.nl/shared/individuals/00380423). To assess the functional impact of the variant, structural analyses using the cryo‐electron microscopy structure of the mammalian polymerase II transcribing complex (PDB ID: 5FLM) (Bernecky et al., 2016) were performed with the VMD software (Humphrey et al., 1996). Different from what was observed for the majority of the pathogenic missense changes, which cluster around the catalytic site, Glu1289 does not map in the sites of the subunit binding to DNA or RNA. The residue, highly conserved among *POLR2A* orthologues (Supplementary Figure S2 a), is located in the region interacting with the transcription‐elongation factor TFIIS, whose function is required for back‐tracking of the enzyme due to elongation blocks or nucleotide misincorporation (Supplementary Figure S2 b and c). In the same region, a recurrent pathogenic amino acid substitution (p.Asn1251Ser) had previously been reported in three individuals (Haijes et al., 2019; Hansen et al., 2021). Moreover, a missense change affecting Glu1230 (p.Glu1230Lys) in the yeast orthologue (corresponding to Asp1249 in *POLR2A*) was shown to reduce the interaction of the subunit with TFIIS (Malagon et al., 2006). Of note, Gly1289 is located at the C‐terminus of a short stretch (Q1270‐V1275) that is not present in yeast and not resolved in the available structure of the complex. It has been suggested that this inserted stretch is relevant for binding to other factors with TFIIS‐like domains (Bernecky et al., 2016). Based on the cryo‐electron microscopy structure of the mammalian complex, the Glu‐to‐Lys substitution was expected to affect the interaction between *POLR2A* and TFIIS (and possibly other partners with TFIIS‐like domains) by disrupting the hydrogen bond involving this residue and Asn56 in TFIIS (Supplementary Figure S2 d), which would result in an increased error and reduced elongation rates (Haijes et al., 2019).

## 4 DISCUSSION

Haijes et al. described 16 individuals (including one aborted fetus) with de novo heterozygous variants in *POLR2A* (10 missense variants, three truncating variants, and three in‐frame deletions), who were documented to be affected by a neurodevelopmental disorder with hypotonia and variable intellectual disability and behavioral abnormalities. In general, compared to loss‐of‐function variants, missense ones were associated with a more severe phenotype characterized by profound infantile‐onset hypotonia and developmental delay. The explanation likely lies in a dominant‐negative effect exerted by the mutated subunit on the function of the holoenzyme, impacting on gene transcription, whereas loss‐of‐function variants leading to the loss of the subunit are expected to result in haploinsufficiency, causing a milder phenotype (Haijes et al., 2019). Hansen et al. have recently provided additional evidence of the pathogenic role of germline *POLR2A* variants, pointing out the multisystemic involvement and the clinical variability of the phenotype of the syndrome in 12 individuals carrying de novo or inherited *POL2RA* variants; several previously unreported phenotypes were also observed (Hansen et al., 2021). Of note, no CNS tumors were reported in both studies.

Consistent with the previously reported findings, the present pediatric case heterozygous for a de novo missense change (p.Glu1289Lys) in *POLR2A* was affected by a severe and progressive form of neurodevelopmental syndrome with hypotonia, cognitive deficits, attention and behavioral disorders, associated with orofacial cleft, urological, skeletal, and facial anomalies. His clinical presentation fits well with the phenotypic spectrum associated with pathogenic *POLR2A* variants (Table 1). The presence of ependymoma as an additional feature, however, expands the awareness about genotype–phenotype correlation regarding *POLR2A* variants, adding new knowledge to both the phenotypic spectrum of *POLR2A* variants and the genetic etiology of ependymomas.

The genetic variant found in our patient is relatively close to p.Asn1251Ser, a variant previously described in three individuals by Hansen et al. (individuals 4 and 5) and Haijes et al. (individual 14). The missense variant affects key residues contributing to the intermolecular binding network of *POLR2A* with the transcription‐elongation factor TFIIS, and is predicted to result in an increased error rate and reduced elongation rates (Haijes et al., 2019). All three patients had a phenotype similarity with our case, showing neurocognitive developmental delay, behavioral disturbances, and facial dysmorphisms. Nevertheless, epilepsy was absent in the present patient. Brain MRI abnormalities, but no CNS tumors, were present (Haijes et al., 2019; Hansen et al., 2021).

Gait ataxia, secondary to the obstructive hydrocephalus, was the presenting sign in our patient. This type of hydrocephalus is due to a blockage of cerebrospinal fluid outflow from the ventricular system to the subarachnoid space, in our case caused by the posterior fossa ependymoma (Lin & Riva‐Cambrin, 2015). Gait ataxia and other signs or symptoms attributable to hydrocephalus may help in the diagnostic workup aiming to detect patients with ependymoma‐associated *POLR2A* variants since obstructive hydrocephalus is present in up to 90% of patients with posterior fossa tumors at diagnosis (Bhatia et al., 2009; Raimondi & Tomita, 1981).

In 2015, Liu et al. showed that *POLR2A* in human cancers is frequently co‐deleted with *TP53*, one of the best‐known human tumor suppressor genes. Given the essential function of *POLR2A*, further suppression by the RNA polymerase inhibitor alpha‐amanitin could inhibit the survival and the proliferation of colorectal cancer cells with hemizygous *TP53* and *POLR2A* deletion, suggesting a novel strategy for molecular targeted therapy (Liu et al., 2015). Clark et al. later discovered a specific subset of benign meningiomas characterized by recurrent mutations in *POLR2A*, confirmed as somatic in all tumor tissue specimens with available blood pairing, for the first time describing an implication of *POLR2A* in human disease. *POLR2A* mutant tumors showed recurrent dock domain mutations affecting both the interaction between RNA polymerase II and transcription factors as well as the subsequent formation of the pre‐initiation complex, necessary for the transcription of protein‐coding genes in eukaryotes (Clark et al., 2016). Moreover, a recent retrospective study reported, especially for WHO grade I skull‐base meningiomas, *POLR2A* pathogenic variants found in tumor samples could be a potentially ideal marker of significantly worse prognosis and a suitable predictor of recurrence (Okano et al., 2021).

Posterior fossa ependymomas lack a signature of recurrent genetic events (Mack et al., 2014), but can be classified according to their epigenetic hallmark into two main groups: group A tumors (EFP‐A) are characterized by the absence of histone H3 K27 trimethylation, whereas group B tumors (EFP‐B) show a high level of trimethylation of histone H3 K27 (Panwalkar et al., 2017). EFP‐A tumors have a worse prognosis, especially in the presence of chromosome 1q gain (Gritsch et al., 2022; Pajtler et al., 2015). DNA methylation profiling is considered a diagnostic tool, useful in challenging cases when ependymoma is included in the differential diagnosis based on anatomic location and histopathologic neoplastic features (Pajtler et al., 2015, 2018; Witt et al., 2018).

The possible causative role of somatic *POLR2A* variants in ependymoma is instead unknown, despite the great advances made in the study of pathogenesis and biological profile of ependymomas, due to the evolution of gene array technology and the recent discovery of novel tumor subgroups based on DNA methylation profiling data (Malbari & Lindsay, 2020). The pathophysiology of the tumorigenic process in an individual carrying a germline *POLR2A* variant is likewise dubious. A number of variants in oncogenes and tumor suppressor genes are traditionally linked to the development of pediatric ependymomas. NF2, MEN1 syndrome, and Turcot syndrome are the best‐known genetic syndromes associated with ependymoma. However, pathogenic variants in the genes related to these familial syndromes (*NF2, MEN1, APC*, respectively) have only been observed in a few cases of sporadic ependymomas (de Bont et al., 2008). The eventual inclusion of the *POLR2A* neurodevelopmental disorder among the cancer predisposition syndromes with possible development of ependymomas is to be considered, although additional evidence is required due to the limited number of patients thus far reported with germline *POLR2A* variants. Nevertheless, the present observation, while acknowledging the possibility of a coincidental relationship between ependymoma and *POLR2A* variant, suggests adding POLR2A to the list of genes to include in oncopediatric next‐generation sequencing panels, especially for patients with ependymoma associated with developmental delay, malformations, and facial anomalies.

## 5 CONCLUSIONS

This is the first report describing an ependymoma as an associated feature of the neurodevelopmental syndrome caused by a germline *POLR2A* variant. Since there is currently scanty information on the clinical variability associated with pathogenic *POLR2A* variants, further studies are required to explore the possibility of a differential clinical and functional impact of the different classes of these variants and their possible contribution to the predisposition to ependymomas inside the germline *POLR2A* neurodevelopmental disorder.

## AUTHOR CONTRIBUTIONS

Roberto Paparella: Conceptualization, Writing – Original Draft. Anna Maria Caroleo: Writing – Original Draft. Emanuele Agolini: Data Curation, Writing – Review & Editing. Giovanni Chillemi: Writing – Review & Editing, Funding Acquisition. Evelina Miele: Data Curation, Investigation. Lucia Pedace: Resources. Martina Rinelli: Data Curation, Investigation. Simone Pizzi: Data Curation. Luigi Boccuto: Supervision, Writing – Review & Editing. Giovanna Stefania Colafati: Resources, Visualization. Mariachiara Lodi: Resources. Antonella Cacchione: Resources, Data Curation. Andrea Carai: Writing – Review & Editing. Maria Cristina Digilio: Writing – Review & Editing. Paolo Tomà: Resources, Visualization. Marco Tartaglia: Writing – Review & Editing. Angela Mastronuzzi: Supervision, Writing – Review & Editing.

## FUNDING INFORMATION

This work was supported in part by funding from the Italian Ministry of Health (RF‐2018‐12366931, to G.C.).

## CONFLICT OF INTEREST

The authors declare that they have no conflict of interest.

## Supporting information


**Supplementary Table S1** Global DNA Methylation calibrated scores of the index case in the top 10 recognized methylation classes according to Brain tumor Classifier v11b4.
Supplementary Figure S1. Copy number variation plot calculated from DNA methylation array data of the tumor sample. Depiction of structural rearrangements involving autosomes and X/Y chromosomes. Gains/amplifications represent positive (green), losses negative (red) deviations from the baseline. Twenty‐nine tumor‐relevant genomic regions are highlighted.
Supplementary Figure S2. Structural impact of p.Glu1289Lys in *POLR2A* on RNA polymerase II function. a) Conservation of the *POLR2A* amino acid encompassing Glu1289 among orthologues. The p.Glu1289Lys variant is highlighted in red. Sites of known mutations in *H. sapiens* and *S. cerevisiae* are highlighted in cyan and green, respectively. b, c) Two rotated visualization of the cryo‐electron microscopy structure of mammalian RNA polymerase II transcribing complex (PDB id 5FLM). The 1246–1300 region of *POLR2A*, TFIIS, DNA and RNA molecules have been rendered in opaque mode while the remaining subunits are visualized in transparent mode. The van der Waals surface of Glu1289 and the other two residues indicated in panel a are visualized using the same colors. (d) Enlargement view of the region of the complex surrounding Glu1289. Lateral chains of Glu1289 and Asn56 (TFIIS) are visualized in licorice mode. The other residues are visualized as van der Waals surfaces (colors as in panel a).

## Data Availability

All data are available in the manuscript or in the Supplementary Information.

## References

[ajmga62869-bib-0001] Bernecky, C. , Herzog, F. , Baumeister, W. , Plitzko, J. M. , & Cramer, P. (2016). Structure of transcribing mammalian RNA polymerase II. Nature, 529(7587), 551–554. 10.1038/nature16482 26789250

[ajmga62869-bib-0002] Bhatia, R. , Tahir, M. , & Chandler, C. L. (2009). The management of hydrocephalus in children with posterior fossa tumours: The role of pre‐resectional endoscopic third ventriculostomy. Pediatric Neurosurgery, 45(3), 186–191. 10.1159/000222668 19494562

[ajmga62869-bib-0003] Cacchione, A. , Lodi, M. , Carai, A. , Miele, E. , Tartaglia, M. , Megaro, G. , Del Baldo, G. , Alessi, I. , Colafati, G. S. , Carboni, A. , Boccuto, L. , Diomedi Camassei, F. , Catanzaro, G. , Po, A. , Ferretti, E. , Pedace, L. , Pizzi, S. , Folgiero, V. , Pezzullo, M. , … Mastronuzzi, A. (2021). Upfront treatment with mtor inhibitor everolimus in pediatric low‐grade gliomas: A single‐center experience. International Journal of Cancer, 148(10), 2522–2534. 10.1002/ijc.33438 33320972

[ajmga62869-bib-0004] Campian, J. , & Gutmann, D. H. (2017). CNS tumors in neurofibromatosis. Journal of Clinical Oncology, 35(21), 2378–2385. 10.1200/JCO.2016.71.7199 28640700 PMC5516481

[ajmga62869-bib-0005] Capper, D. , Jones, D. T. W. , Sill, M. , Hovestadt, V. , Schrimpf, D. , Sturm, D. , Koelsche, C. , Sahm, F. , Chavez, L. , Reuss, D. E. , Kratz, A. , Wefers, A. K. , Huang, K. , Pajtler, K. W. , Schweizer, L. , Stichel, D. , Olar, A. , Engel, N. W. , Lindenberg, K. , … Pfister, S. M. (2018). DNA methylation‐based classification of central nervous system tumours. Nature, 555(7697), 469–474. 10.1038/nature26000 29539639 PMC6093218

[ajmga62869-bib-0006] Clark, V. E. , Harmancı, A. S. , Bai, H. , Youngblood, M. W. , Lee, T. I. , Baranoski, J. F. , Ercan‐Sencicek, A. G. , Abraham, B. J. , Weintraub, A. S. , Hnisz, D. , Simon, M. , Krischek, B. , Erson‐Omay, E. Z. , Henegariu, O. , Carrión‐Grant, G. , Mishra‐Gorur, K. , Durán, D. , Goldmann, J. E. , Schramm, J. , … Günel, M. (2016). Recurrent somatic mutations in POLR2A define a distinct subset of meningiomas. Nature Genetics, 48(10), 1253–1259. 10.1038/ng.3651 27548314 PMC5114141

[ajmga62869-bib-0007] de Bont, J. M. , Packer, R. J. , Michiels, E. M. , den Boer, M. L. , & Pieters, R. (2008). Biological background of pediatric medulloblastoma and ependymoma: A review from a translational research perspective. Neuro‐Oncology, 10(6), 1040–1060. 10.1215/15228517-2008-059 18676356 PMC2719002

[ajmga62869-bib-0026] Gritsch, S. , Batchelor, T. T. , & Gonzalez Castro, L. N. (2022). Diagnostic, therapeutic, and prognostic implications of the 2021 World Health Organization classification of tumors of the central nervous system. Cancer, 128(1), 47–58. 10.1002/cncr.33918 34633681

[ajmga62869-bib-0009] Haijes, H. A. , Koster, M. J. E. , Rehmann, H. , Li, D. , Hakonarson, H. , Cappuccio, G. , Hancarova, M. , Lehalle, D. , Reardon, W. , Schaefer, G. B. , Lehman, A. , van de Laar, I. M. B. H. , Tesselaar, C. D. , Turner, C. , Goldenberg, A. , Patrier, S. , Thevenon, J. , Pinelli, M. , Brunetti‐Pierri, N. , … van Hasselt, P. M. (2019). De novo heterozygous POLR2A variants cause a neurodevelopmental syndrome with profound infantile‐onset hypotonia. The American Journal of Human Genetics, 105(2), 283–301. 10.1016/j.ajhg.2019.06.016 31353023 PMC6699192

[ajmga62869-bib-0010] Hansen, A. W. , Arora, P. , Khayat, M. M. , Smith, L. J. , Lewis, A. M. , Rossetti, L. Z. , Jayaseelan, J. , Cristian, I. , Haynes, D. , DiTroia, S. , Meeks, N. , Delgado, M. R. , Rosenfeld, J. A. , Pais, L. , White, S. M. , Meng, Q. , Pehlivan, D. , Liu, P. , Gingras, M.‐C. , … Gibbs, R. A. (2021). Germline mutation in POLR2A: A heterogeneous, multi‐systemic developmental disorder characterized by transcriptional dysregulation. HGG Advances, 2(1), 100014. 10.1016/j.xhgg.2020.100014 33665635 PMC7928427

[ajmga62869-bib-0011] Humphrey, W. , Dalke, A. , & Schulten, K. (1996). VMD: Visual molecular dynamics. Journal of Molecular Graphics, 14(1), 33–38. 10.1016/0263-7855(96)00018-5 8744570

[ajmga62869-bib-0012] Kilday, J.‐P. , Rahman, R. , Dyer, S. , Ridley, L. , Lowe, J. , Coyle, B. , & Grundy, R. (2009). Pediatric ependymoma: Biological perspectives. Molecular Cancer Research, 7(6), 765–786. 10.1158/1541-7786.MCR-08-0584 19531565

[ajmga62869-bib-0013] Lin, C.‐T. , & Riva‐Cambrin, J. K. (2015). Management of posterior fossa tumors and hydrocephalus in children: A review. Child's Nervous System, 31(10), 1781–1789. 10.1007/s00381-015-2781-8 26351230

[ajmga62869-bib-0014] Liu, Y. , Zhang, X. , Han, C. , Wan, G. , Huang, X. , Ivan, C. , Jiang, D. , Rodriguez‐Aguayo, C. , Lopez‐Berestein, G. , Rao, P. H. , Maru, D. M. , Pahl, A. , He, X. , Sood, A. K. , Ellis, L. M. , Anderl, J. , & Lu, X. (2015). TP53 loss creates therapeutic vulnerability in colorectal cancer. Nature, 520(7549), 697–701. 10.1038/nature14418 25901683 PMC4417759

[ajmga62869-bib-0015] Mack, S. C. , Witt, H. , Piro, R. M. , Gu, L. , Zuyderduyn, S. , Stütz, A. M. , Wang, X. , Gallo, M. , Garzia, L. , Zayne, K. , Zhang, X. , Ramaswamy, V. , Jäger, N. , Jones, D. T. W. , Sill, M. , Pugh, T. J. , Ryzhova, M. , Wani, K. M. , Shih, D. J. H. , … Taylor, M. D. (2014). Epigenomic alterations define lethal CIMP‐positive ependymomas of infancy. Nature, 506(7489), 445–450. 10.1038/nature13108 24553142 PMC4174313

[ajmga62869-bib-0016] Malagon, F. , Kireeva, M. L. , Shafer, B. K. , Lubkowska, L. , Kashlev, M. , & Strathern, J. N. (2006). Mutations in the *Saccharomyces cerevisiae RPB1* gene conferring hypersensitivity to 6‐Azauracil. Genetics, 172(4), 2201–2209. 10.1534/genetics.105.052415 16510790 PMC1456368

[ajmga62869-bib-0017] Malbari, F. , & Lindsay, H. (2020). Genetics of common pediatric brain tumors. Pediatric Neurology, 104, 3–12. 10.1016/j.pediatrneurol.2019.08.004 31948735

[ajmga62869-bib-0018] Okano, A. , Miyawaki, S. , Hongo, H. , Dofuku, S. , Teranishi, Y. , Mitsui, J. , Tanaka, M. , Shin, M. , Nakatomi, H. , & Saito, N. (2021). Associations of pathological diagnosis and genetic abnormalities in meningiomas with the embryological origins of the meninges. Scientific Reports, 11(1), 6987. 10.1038/s41598-021-86298-9 33772057 PMC7998008

[ajmga62869-bib-0019] Pajtler, K. W. , Wen, J. , Sill, M. , Lin, T. , Orisme, W. , Tang, B. , Hübner, J.‐M. , Ramaswamy, V. , Jia, S. , Dalton, J. D. , Haupfear, K. , Rogers, H. A. , Punchihewa, C. , Lee, R. , Easton, J. , Wu, G. , Ritzmann, T. A. , Chapman, R. , Chavez, L. , … Ellison, D. W. (2018). Molecular heterogeneity and CXorf67 alterations in posterior fossa group a (PFA) ependymomas. Acta Neuropathologica, 136(2), 211–226. 10.1007/s00401-018-1877-0 29909548 PMC6105278

[ajmga62869-bib-0020] Pajtler, K. W. , Witt, H. , Sill, M. , Jones, D. T. W. , Hovestadt, V. , Kratochwil, F. , Wani, K. , Tatevossian, R. , Punchihewa, C. , Johann, P. , Reimand, J. , Warnatz, H.‐J. , Ryzhova, M. , Mack, S. , Ramaswamy, V. , Capper, D. , Schweizer, L. , Sieber, L. , Wittmann, A. , … Pfister, S. M. (2015). Molecular classification of ependymal tumors across all CNS compartments, histopathological grades, and age groups. Cancer Cell, 27(5), 728–743. 10.1016/j.ccell.2015.04.002 25965575 PMC4712639

[ajmga62869-bib-0021] Panwalkar, P. , Clark, J. , Ramaswamy, V. , Hawes, D. , Yang, F. , Dunham, C. , Yip, S. , Hukin, J. , Sun, Y. , Schipper, M. J. , Chavez, L. , Margol, A. , Pekmezci, M. , Chung, C. , Banda, A. , Bayliss, J. M. , Curry, S. J. , Santi, M. , Rodriguez, F. J. , … Venneti, S. (2017). Immunohistochemical analysis of H3K27me3 demonstrates global reduction in group: A childhood posterior fossa ependymoma and is a powerful predictor of outcome. Acta Neuropathologica, 134(5), 705–714. 10.1007/s00401-017-1752-4 28733933 PMC5647236

[ajmga62869-bib-0022] Raimondi, A. J. , & Tomita, T. (1981). Hydrocephalus and infratentorial tumors: Incidence, clinical picture, and treatment. Journal of Neurosurgery, 55(2), 174–182. 10.3171/jns.1981.55.2.0174 7252539

[ajmga62869-bib-0023] Shu, H.‐K. G. , Sall, W. F. , Maity, A. , Tochner, Z. A. , Janss, A. J. , Belasco, J. B. , Rorke‐Adams, L. B. , Phillips, P. C. , Sutton, L. N. , & Fisher, M. J. (2007). Childhood intracranial ependymoma: Twenty‐year experience from a single institution. Cancer, 110(2), 432–441. 10.1002/cncr.22782 17559078

[ajmga62869-bib-0024] Wintzerith, M. , Acker, J. , Vicaire, S. , Vigneron, M. , & Kedinger, C. (1992). Complete sequence of the human RNA polymerase II largest subunit. Nucleic Acids Research, 20(4), 910–910. 10.1093/nar/20.4.910 1542581 PMC312037

[ajmga62869-bib-0025] Witt, H. , Gramatzki, D. , Hentschel, B. , Pajtler, K. W. , Felsberg, J. , Schackert, G. , Löffler, M. , Capper, D. , Sahm, F. , Sill, M. , von Deimling, A. , Kool, M. , Herrlinger, U. , Westphal, M. , Pietsch, T. , Reifenberger, G. , Pfister, S. M. , Tonn, J. C. , Weller, M. , & Network, G. G. (2018). DNA methylation‐based classification of ependymomas in adulthood: Implications for diagnosis and treatment. Neuro‐Oncology, 20(12), 1616–1624. 10.1093/neuonc/noy118 30053291 PMC6231197

