# Supplementary material for: Posterior fossa ependymoma in neurodevelopmental syndrome caused by a de novo germline pathogenic POLR2A variant
Source: Am J Med Genet A. 2022 Jun 11;188(9):2796–802. doi: 10.1002/ajmg.a.62869 (PMC9543264; doi:10.1002/ajmg.a.62869)

**Supplementary Table S1.** Global DNA Methylation calibrated scores of the index case in the top ten recognized methylation classes according to Brain tumor Classifier v11b4.

| **Methylation class Brain tumor Classifier v11b4** | **Calibrated score** |
| --- | --- |
| EPN_PF_A | 0.999187301 |
| DMG_K27 | 5.39E-05 |
| MB_SHH_CHL_AD | 5.19E-05 |
| PLEX_PED_B | 4.06E-05 |
| SUBEPN_PF | 3.47E-05 |
| PLEX_PED_A | 3.28E-05 |
| MB_G3 | 2.80E-05 |
| MNG | 2.75E-05 |
| MB_G4 | 2.45E-05 |
| GBM_MES | 2.15E-05 |

EPN_PF_A: ependymoma, posterior fossa group A; DMG_K27: diffuse midline glioma H3 K27M mutant; MB_SHH_CHL_AD: medulloblastoma, subclass SHH A (children and adult); PLEX_PED_B: plexus tumor, pediatric subtype B; SUBEPN_PF: subependymoma, posterior fossa; PLEX_PED_A: plexus tumor, pediatric subtype A; MB_G3: medulloblastoma, subclass group 3; MNG: meningioma; MB_G4: medulloblastoma, subclass group 4; GBM_MES: glioblastoma, IDH wildtype, subclass mesenchymal

**Supplementary Figure S1.** Copy number variation plot calculated from DNA methylation array data of the tumor sample. Depiction of structural rearrangements involving autosomes and X/Y chromosomes. Gains/amplifications represent positive (green), losses negative (red) deviations from the baseline. Twenty-nine tumor-relevant genomic regions are highlighted.


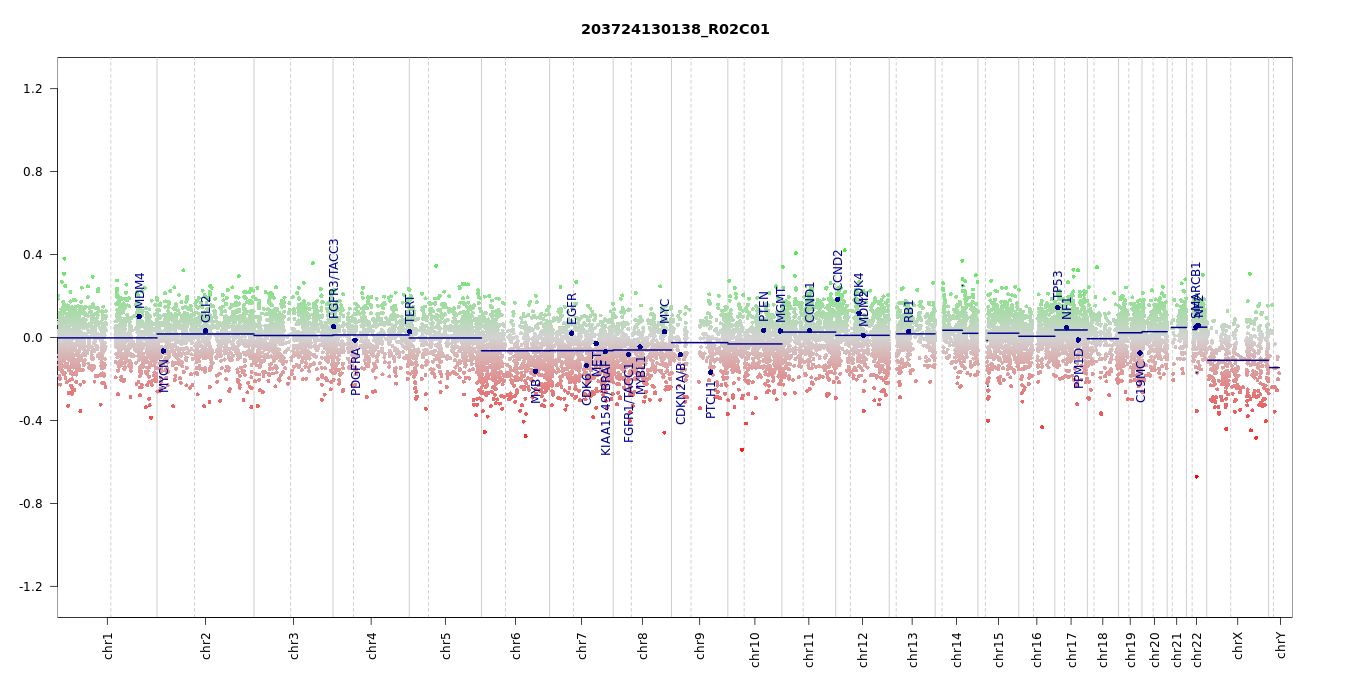


**Supplementary Figure S2.** Structural impact of p.Glu1289Lys in *POLR2A* on RNA polymerase II function. **a)** Conservation of the *POLR2A* amino acid encompassing Glu1289 among orthologues. The p.Glu1289Lys variant is highlighted in red. Sites of known mutations in *H. sapiens* and *S. cerevisiae* are highlighted in cyan and green, respectively. **b, c)** Two rotated visualization of the cryo-electron microscopy structure of mammalian RNA polymerase II transcribing complex (PDB id 5FLM). The 1246-1300 region of *POLR2A*, TFIIS, DNA and RNA molecules have been rendered in opaque mode while the remaining subunits are visualized in transparent mode. The van der Waals surface of Glu1289 and the other two residues indicated in panel **a** are visualized using the same colors. **d)** Enlargement view of the region of the complex surrounding Glu1289. Lateral chains of Glu1289 and Asn56 (TFIIS) are visualized in licorice mode. The other residues are visualized as van der Waals surfaces (colors as in panel **a**).


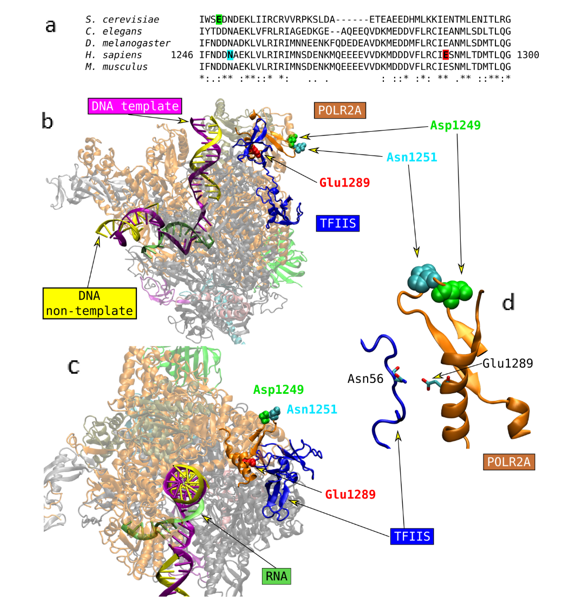

Supplement: Supplementary file 1 — Supplementary Table S1 Global DNA Methylation calibrated scores of the index case in the top 10 recognized methylation classes according to Brain tumor Classifier v11b4. Supplementary Figure S1. Copy number variation plot calculated from DNA methylation array data of the tumor sample. Depiction of structural rearrangements involving autosomes and X/Y chromosomes. Gains/amplifications represent positive (green), losses negative (red) deviations from the baseline. Twenty‐nine tumor‐relevant genomic regions are highlighted. Supplementary Figure S2. Structural impact of p.Glu1289Lys in POLR2A on RNA polymerase II function. a) Conservation of the POLR2A amino acid encompassing Glu1289 among orthologues. The p.Glu1289Lys variant is highlighted in red. Sites of known mutations in H. sapiens and S. cerevisiae are highlighted in cyan and green, respectively. b, c) Two rotated visualization of the cryo‐electron microscopy structure of mammalian RNA polymerase II transcribing complex (PDB id 5FLM). The 1246–1300 region of POLR2A, TFIIS, DNA and RNA molecules have been rendered in opaque mode while the remaining subunits are visualized in transparent mode. The van der Waals surface of Glu1289 and the other two residues indicated in panel a are visualized using the same colors. (d) Enlargement view of the region of the complex surrounding Glu1289. Lateral chains of Glu1289 and Asn56 (TFIIS) are visualized in licorice mode. The other residues are visualized as van der Waals surfaces (colors as in panel a). [file AJMG-188-2796-s001.docx]
